# Transcriptome and targeted hormone metabolome reveal the molecular mechanisms of flower abscission in camellia

**DOI:** 10.3389/fpls.2022.1076037

**Published:** 2022-12-22

**Authors:** Yanfei Cai, Jing Meng, Yinshan Cui, Min Tian, Ziming Shi, Jihua Wang

**Affiliations:** ^1^ Flower Research Institute of Yunnan Academy of Agricultural Sciences, Kunming, Yunnan, China; ^2^ National Engineering Research Center for Ornamental Horticulture, Kunming, Yunnan, China; ^3^ College of Horticulture and Landscape, Yunnan Agricultural University, Kunming, Yunnan, China; ^4^ Yunnan Pulis Biotechnology Co. Ltd., Kunming, Yunnan, China

**Keywords:** camellia, flower abscission, phytohormones, transcription factors, molecular mechanisms

## Abstract

**Introduction:**

Camellia is among the most ornamentally valuable flowers and plants worldwide. Flower abscission typically causes significant financial losses by the horticultural landscape. Previous research has revealed that phytohormones, transcription factors, and other genes involved in floral development regulate the maintenance and mortality of flowers

**Methods:**

In this study, for the first time, the transcriptomes and targeted hormone metabolomics of three developmental stages of the receptacles of two distinct camellia strains (CF: abscission strain, CHF: nonabscission strain) were analyzed to determine their roles in regulating blossom abscission in camellia.

**Results:**

ABA content was shown to be considerably upregulated throughout all phases of CF development, as were the genes implicated in the ABA production pathway and their downstream counterparts. Highly expressed genes in CF were involved in galactose metabolism, phenylpropanoid biosynthesis, amino and nucleotide sugar metabolism, pentose and glucuronate interconversions, and MAPK. Among others, highly expressed genes in CHF are associated with fructose and mannose metabolism, alpha-linolenic acid metabolism, biosynthesis of secondary metabolites, starch and sucrose metabolism, and cutin, suberin, and wax biosynthesis. A vast variety of stress response-related pathways and redox-related activities were also shown to be active in CHF. In contrast, CF dramatically activated pathways associated with lignin production, keratinogenesis, cell wall biogenesis, and ABA response. A comparative transcriptomic study of the CF and CHF pathways revealed that the downstream response pathways of hormones, including CTK, BR, IAA, ethylene, and GA, were very active in CF, indicating a significant amount of signal transduction and transcriptional regulation by CF. In addition, members of the transcription factor family, such as MYB, bHLH, MADS, and WD40, may regulate flower abscission.

**Discussion:**

A comparative transcriptome analysis of two distinct strains of camellia receptacles elucidates the molecular processes and regulatory characteristics of flower abscission and provides direction for the targeted improvement and breeding of camellia.

## Introduction

Camellia is a genus of flowering plants in the Camellia family that is extensively dispersed throughout Southeast Asia, from the Himalayas to Japan and from southern China (Guangxi and Yunnan) to Sumatra (Zhang et al., 2012; Su et al., 2014). Several species of Camellia are economically significant. The bright camellias of *C. japonica*, *C. reticulata*, *C. saluenensis*, and *C. sasanqua* are well known. Ornamental camellia (Chinese camellia) has been cultivated in China for 2,000 years; common camellia was introduced to Japan over 1,000 years ago; and ornamental camellia was introduced to Europe and the Americas in the late 1,870s; it is now a popular flowering and landscape shrub throughout the world. There are currently more than 30,000 ornamental camellia cultivars produced around the world. Despite this, camellias are frequently plagued by flower abscission, resulting in substantial economic losses (Zhang et al., 2022).

Abscission is a ubiquitous phenomenon that occurs at numerous phases of the life cycle of plants (Patharkar and Walker, 2018). Fall foliage and fruit loss after maturity are classic examples, as are organ damage and infection-induced losses. Controlling abscission is still important in agriculture and horticulture, as many crops, including citrus, cotton, Brassica, and certain rice cultivars, suffer production losses owing to abscission. The abscission zone (AZ) is a specialized cell layer where cell separation is assisted by hydrolases during abscission (Phetsirikoon et al., 2016; Merelo et al., 2017). The abscission of buds, branches, petioles, leaves, flowers, and fruits by plants is influenced by environmental conditions such as temperature, light quality, disease, water stress, and nutrition (Lee et al., 2021; Lu et al., 2021; Dutta et al., 2022). The generation of AZ is governed by plant hormones such as auxin, abscisic acid, jasmonic acid, and ethylene (Sriskantharajah et al., 2021; Dziurka et al., 2022), as well as by genes involved in the process of organ separation, such as BLADE ON PETIOLE (BOP), HAESA (HAE), HAESA-LIKE2 (HSL2), and CAST AWAY (CST) (Santiago et al., 2016; Meir et al., 2019). Abscission is related to the activity of enzymes such polygalacturonases, xyloglucan endotransglucosylases/hydrolases, -1,4-glucanases/cellulases, and expansins (EXP) (Sriskantharajah et al., 2021; Ma et al., 2021; Wilmowicz et al., 2022). Consequently, variations in the amounts of plant hormones and gene expression may be among the most fundamental factors influencing variability in plant organ abscission.

Abscission inhibitors include the plant growth regulators auxin (IAA) and gibberellin (GA) (Sriskantharajah et al., 2021; Dutta et al., 2022). The finding that IAA reduces ethylene production and signaling, in AZ-A explains why it inhibits abscission (Dong et al., 2021). Similar to endogenous GA, the use of exogenous GA may prevent fruit drop in citrus (Lal et al., 2015; Rokaya et al., 2016). Instead, ethylene, abscisic acid (ABA), and methyl jasmonate (MJ) have been shown to enhance shedding in latest research (Xu et al., 2020; Hewitt et al., 2021; Zhou et al., 2022). Citrus fruit abscission is aided by ethylene, which research shows is concentrated in the abscission zone (Dutta et al., 2022). The abscission zone of apple dropped fruit showed elevated transcript levels of genes involved in ethylene production, including ACS5A and ACO1, as well as ethylene receptor genes, ETR2 and ERS2 (Li et al., 2010). Increased expression of genes such SAMS, ACS, ACO, and ETR in the abscission zone has been linked to the early induction of fruit abscission in melon (Corbacho et al., 2013). The abscission of apple fruits may also be stimulated by the external treatment of ABA. There is a correlation between ABA levels and the formation of senescence and seed dormancy (Vishal and Kumar, 2018), and ABA levels tend to rise as fruit ripens. As a regulator of ACC levels, ABA may promote shedding by stimulating ethylene biosynthesis (Kou et al., 2021; Pattyn et al., 2021). Overall, the present study has examined the connection between phytohormones and plant organ shedding, however the underlying molecular process is unclear.

The transcriptomes and targeted hormone metabonomics of three developmental stages of the receptacles of two distinct camellia strains were investigated to understand their involvement in regulating blossom abscission in camellia in this study. The objective was to determine the differences between CF and CHF with respect to flower development, as well as the molecular pathways and key genes that control camellia flower abscission. At various phases of development, CF and CHF demonstrated significant differences in metabolic pathways, hormone content, and regulatory factor expression, as determined by our investigation. These results suggest potential molecular explanations and research directions with respect to the floral abscission phenotypes of CF and CHF.

## Material and methods

### Plant materials and samples collected

**Figure 1:**
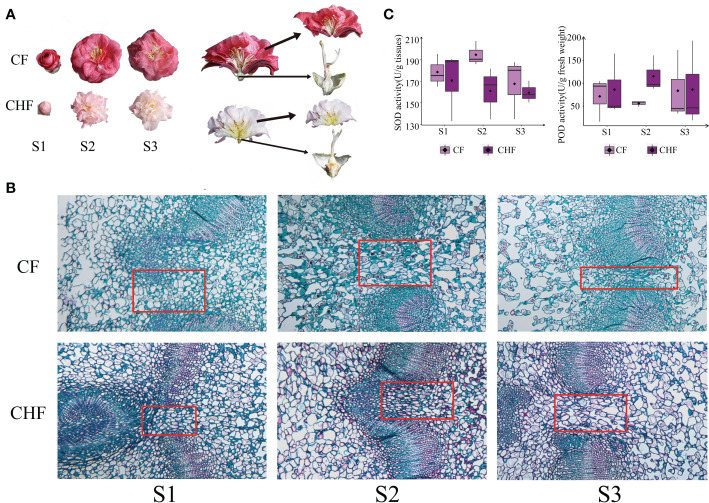
Morphology and enzymatic activity assessment of camellia. **(A)** The receptacles and petals of CF and CHF flowers are depicted individually. **(B)** Histological structure of *Camellia* at different stages at a final magnification of 400X. The red box is where the representative structure sites of the abscission features. **(C)** Determination of SOD and POD enzymatic activity at the S1, S2, and S3 phases of CF and CHF. There was no significant difference among different stages.

Two different varieties of Camellia, Camellia ‘Flowerwood’ (CF) and Camellia ‘High Fragrance’ (CHF) were used for the experiment. Details of two varieties of Camellia can be found at https://camellia.iflora.cn/. All plants were grown in greenhouses at International Flora Technology Innovation Center (Kunming, China). The material was propagated by branch cuttings and planted in flowerpot soil for 3 years. CF is the easy abscission strain; CHF is the difficult abscission strain. Both strains were originally developed by our team, and during the growing process, it was found that the petals had fallen off. CF showed easier petal fall and abscission during earlier developmental stages due to the presence of larger petals than those of CHF **(** **Figure 1A** **)**. All pedicel samples were collected from January 10 to February 3, 2022. Samples were selected based on the degree of abscission, with S1 defined as no abscission at all, S2 as partial abscission, and S3 as complete abscission. The receptacles were carefully peeled off and then quickly placed in liquid nitrogen for freezing and storage until the next experiment.

### Antioxidant enzyme activity measurements

Spectrophotometry was used to determine the activities of superoxide dismutase (SOD) and peroxidase (POD). The samples were ground in liquid nitrogen and used to prepare extracts. One gram of the powder was added to 9 mL of sodium phosphate buffer (100 mM, pH 7.0), vortexed and mixed at 3,500 rpm/min, and centrifuged for 10 minutes to obtain the supernatant as a 10% homogenate supernatant for the SOD and POD analyses. The activity of SOD was measured by nitroblue tetrazolium reduction, and POD activity was measured by the guaiacol oxidation process according to (González et al., 2021). The absorbance was measured at 450 nm (A450 nm) using a microplate reader. In this reaction system, the corresponding enzyme amount when the SOD inhibition rate reached 50% was one SOD activity unit (U). The POD activity was measured by measuring the absorbance at 420 nm. One unit (1 U) of POD enzymatic activity was defined as the amount of enzyme catalyzing 1 μg of substrate per minute per gram of tissue at 37°C. Three technical replicates were performed for the determinations.

### Characterization of the histological structure of Camellia

Different stages (as mentioned above S1, S2 and S3) of pedicel were collected and fixed for 48 h at room temperature in 50% formaldehyde-acetic acid-ethanol (FAA) fixative. Samples were dehydrated in ethanol and xylene and embedded in paraffin. Sections 8 µm thick were cut with a rotary microtome (Leica) and stained with Safranine O-Fast Green for examination under an Olympus microscope.

### Transcriptome sequencing and analysis

Total RNA was extracted from each sample using an RNAprep Pure Plant Plus Kit (Tiangen, China). mRNA was obtained with oligo(Santiago et al.) beads and reverse transcribed to cDNA. The library was then constructed using the NEBNext^®^ Ultra™ RNA Library Prep Kit for Illumina^®^ (NEB, USA) according to the manufacturer’s instructions. Qualified transcriptome libraries were paired-end sequenced on the Illumina HiSeq6000 sequencing platform.

The clean reads were obtained after filtering the raw data. Because there was no available reference genome, all sequences were assembled *de novo* by the Trinity program. The unigenes obtained from the assembly sequences were subsequently clustered using Corset (version 4.6) software. BUSCO software was used to evaluate the splicing quality of unigene sequences. The unigenes were functionally annotated using 7 databases: Nr, Nt, Pfam, KOG/COG, Swiss-Prot, KEGG and GO. Each sample’s reads were mapped to a collection of reference transcripts using the Bowtie aligner (Langmead and Salzberg, 2012), and the amount of each transcript was estimated using RSEM (part of the Trinity package) with the default parameters for paired reads (Li and Dewey, 2011). The level of expression was calculated at both the unigene and isoform levels. Differentially expressed genes (DEGs) between the two groups were identified by the DESeq2 (v1.30.1) package of the R program (v4.0.4). Adjustments were made to the derived P values using the Benjamini−Hochberg method. The DEG threshold was a P-adjusted value <0.05 & |log2(fold change)|>1. The functional analysis of DEGs was performed by enrichment of the GO and KEGG databases using the clusterprofile (v3.18.1) R package.

### Extraction of metabolites and UPLC

Frozen plant samples were ground into powder (30 Hz, 1 min). Fifty milligrams of powder were dissolved in 1 mL methanol/water/formic acid (15:4:1, V/V/V). Ten microliters of internal standard mixed solution (100 ng/mL) was added to each extract as internal standards (IS) for quantification. Each mixture was vortexed for 10 minutes and then centrifuged for 5 min (12,000 r/min at 4°C). Each supernatant was transferred to a microtube, evaporated to dryness, dissolved in 100 μL 80% methanol (V/V), and filtered through a 0.22 μm membrane filter for further LC−MS/MS analysis.

The sample extracts were analyzed using a UPLC−ESI−MS/MS system. Phytohormones were analyzed using scheduled multiple reaction monitoring (MRM). Mass spectrometer parameters, including the declustering potentials (DP) and collision energies (CE) for individual MRM transitions, were performed with further DP and CE optimization. A specific set of MRM transitions was monitored for each period according to the metabolites eluted within this period.

### Bioinformatics analysis of metabolites

Data acquisition was performed using Analyst 1.6.3 software (Sciex). Multiquant 3.0.3 software (Sciex) was used to quantify all metabolites. Unsupervised principal component analysis (PCA) was performed using the R package prcomp (v3.18.1). VIP >= 1 and absolute Log2FC (fold change) >= 1 suggested that metabolites were highly regulated between groups. Using the R tool MetaboAnalyzerR (v3.3.0), VIP values were extracted from the OPLS-DA results, which also included score graphs and permutation plots. The data were log transformed (log2) and mean-centred prior to OPLS-DA, linking annotated metabolites to the KEGG Pathway database. Then, pathways with significantly regulated metabolites were fed into MSEA (metabolite set enrichment analysis), and their significance was determined using the hypergeometric test p values.

### Quantitative real-time polymerase chain reaction (qRT-PCR) analysis

Mastermixes for the qRT-PCR reactions included SYBR Green I Supermix (TaKaRa, Dalian, China) and the specific forward/reverse primers (**Table S1**). The RT-qPCR was run on the qTower 3 g Real-Time PCR Instrument (Analytik Jena, Jena, Germany) with the following cycling conditions: 3 min of predenaturation at 95°C, then 94°C for 10 s, 60°C for 30 s, and 72°C for 90 s (39 cycles). The housekeeping gene Actin was used as an internal control to calculate relative gene expression in this study. All procedures were performed on three independent biological and technical repeats.

## Results

### Morphological identification and activity of SOD and POD

In this study, the camellia varieties CF and CHF were chosen for their unique flower morphologies. CF is distinguished by its large, easily detachable flowers. CHF, on the other hand, has smaller, relatively abscission-resistant blossoms. This study identified the histological structure of the receptacles of CF and CHF flowers at different developmental phases **(** **Figure 1A** **)**. The results of the staining showed that Camellia pedicels of both species became lax with developmental stages. The fenestrated tissues of the pedicels developed pores earlier in CF, and the number of pores was highest in the S3 stages **(** **Figure 1B** **)**. We postulated that the difference in abscission features between CF and CHF may be related to antioxidant levels on the torus; thus, we evaluated the SOD and POD enzyme activities in all samples. During the S2 and S3 stages, CF displayed more SOD enzymatic activity than CHF, but the reverse was true for POD levels. This highlights changes in redox-related enzyme activity between CF and CHF at the receptacle and may possibly represent differences in ROS concentrations at these sites **(** **Figure 1C** **)**.

### Transcriptome differences and enrichment analysis

Transcriptome sequencing yielded a total of 111.51 Gb of available sequences, and a total of 138,557 clustered unigenes were obtained for the *de novo* assembly with an N50 of 1,463. The completeness of the assembly was 77.7% as assessed by BUSCO software, indicating a high quality of the assembly. A large number of unique genes were annotated for subsequent use (**Table S2**).

**Figure 2:**
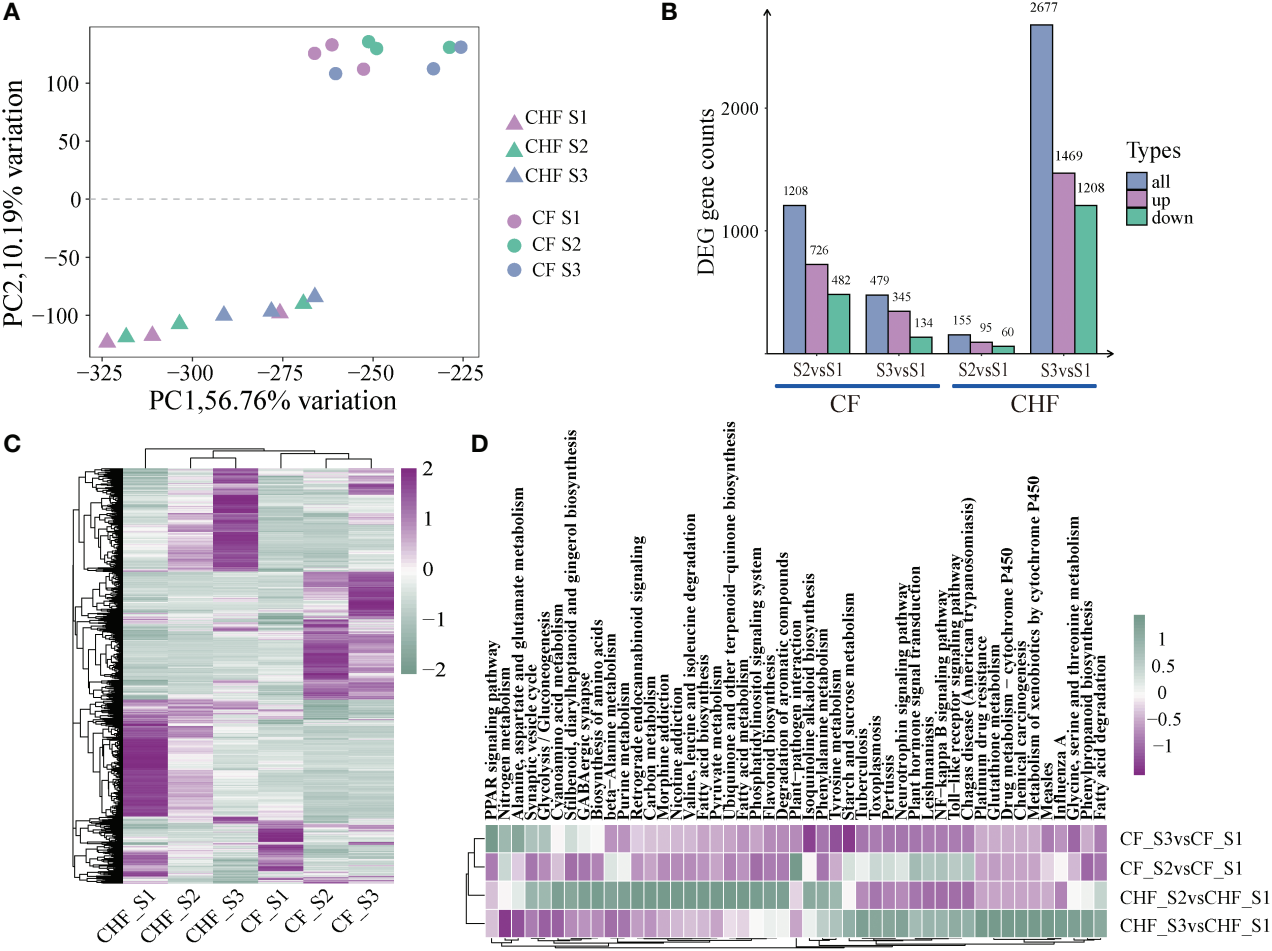
An overview of the RNA-seq data for CF and CHF during the S1, S2, and S3 phases. **(A)** PCA of RNA-seq data showed differences in lines and stages. **(B)** The number of DEGs during different stages for CF and CHF. **(C)** Heatmap showing the global expression of all DEGs. **(D)** KEGG enrichment analysis findings of DEGs among various grouped comparisons, with each hue representing the normalized -log10 value (p).

Morphology and SOD/POD enzymatic activity experiments suggested that substantial variations may exist between CF and CHF developmental phases. To investigate the molecular processes behind their distinct phenotypes, we undertook a comparative transcriptome investigation of three phases of CF and CHF development (S1, S2, S3). All subgroups had excellent within-group repeatability and between-group variability, as determined by PCA between samples **(** **Figure 2A** **).** Comparing S1 and S2 and S1 and S3 of CF revealed 1,208 and 479 differentially expressed genes, respectively, as found by differential analysis. Comparing S1 and S2 and S1 and S3 in CHF, 155 and 2,677 differentially expressed genes were found. This reveals a striking contrast in the dynamics of the transcriptomes of CF and CHF throughout flower development, with CF exhibiting the most dramatic transcriptional alterations at the S2 stage and CHF at the S3 stage. The huge difference in the number of differential genes may imply that CF has quicker flower growth and a shorter floral cycle than CHF, and the rapid flower development of CF may serve a role in its effortless abscission **(** **Figure 2B** **)**. Similarly, the global expression heatmap of DEGs revealed a continuously changing pattern, with CHF exhibiting moderate alterations in the transcriptome during the S2 stage and significant changes during the S3 stage **(** **Figure 2C** **).** CF, on the other hand, displayed sustained dynamic transcriptome alterations throughout both S2 and S3 phases.

Compared with the S1 stage, the DEGs of CF and CHF in the S2 stage were mostly concentrated in metabolic pathways, including plant hormone signal transduction, starch and sucrose metabolism, and phenylpropanoid biosynthesis, indicating that CF is engaged in cell metabolism at this stage. Similarly, the DEGs of CF and CHF at the S3 stage were mostly enriched in metabolic pathways, including flavonoid biosynthesis, fatty acid metabolism, and pyruvate metabolism **(** **Figure 2D** **).** This indicates that CHF is undergoing a metabolic process at this point and reveals that CF and CHF have distinct primary flower development phases. HCHF develops and matures rapidly during the S2 stage, while CHF starts flower development in the S3 stage after S2.

### Expression clustering and functional analysis

**Figure 3:**
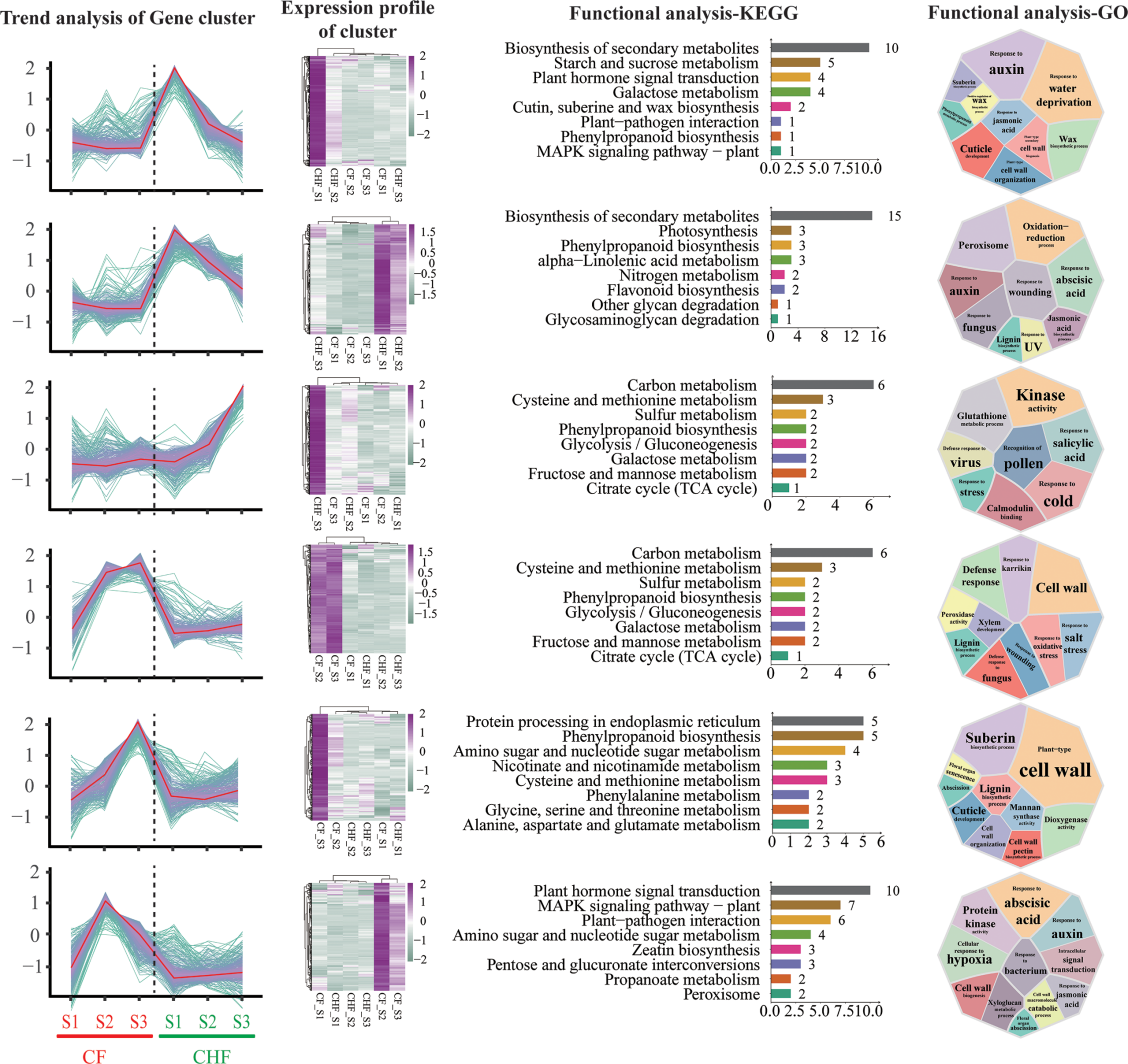
K-means cluster analysis of differentially expressed genes during the S1, S2, and S3 phases for CF and CHF. The first column depicts the expression change trend of DEGs in each module, while the second column displays the expression heatmap of the module’s genes. The third column shows the KEGG functional analysis of the module’s genes, and the fourth column presents the GO functional analysis of the module’s genes.

The k-means clustering approach was utilized to conduct cluster analysis on all DEGs since the dynamic changes in expression profiles at distinct developmental stages across different strains in this research adhered to the features of time series data to a certain degree. The study yielded a total of 12 DEG subgroups (**Figure S1**), and we detected 6 DEG subgroups (subclasses 1, 3, 4, 8, 9, 12) with opposing or distinct patterns between CF and CHF **(** **Figure 3** **).** The functional enrichment analysis of the subgroup genes revealed that many were engaged in cell wall synthesis, stress response, and redox-related pathways, such as cold response, galactose metabolism, and plant-type cell wall organization, among others. These genes are associated with plant hormone signal transduction, cutin, suberin, and wax production, phenylpropanoid biosynthesis, and the MAPK signaling pathway, which suggests that CHF may undergo a process during the S1 stage. GO analysis also revealed that a substantial number of Cluster 1 genes were involved in cell wall-related processes, such as cuticle formation, plant-type secondary cell wall biosynthesis, and positive regulation of the wax biosynthetic process. This shows that Cluster 1 genes are the most transcriptionally active genes in CHF during the S1 stage. During the S1 and S3 periods of CHF flower development, Cluster 3 and Cluster 4 exhibited very high transcriptional activity. Several activities of Cluster 3 genes, including Response to wounding, Oxidation−reduction process, Response to UV, and Response to auxin, are associated with adversity stress. This also shows that CHF is more resistant to both biotic and abiotic stressors than CF. The majority of Cluster 4 genes were closely associated with metabolic pathways, including carbon metabolism, cysteine and methionine metabolism, glycolysis/gluconeogenesis, fructose and mannose metabolism, and galactose metabolism. This demonstrates the active transcriptional activity of CHF during the S3 stage, as well as the significance of the S3 stage in the flower development of CHF. Clusters 8, 9, and 12 are functional modules with significant levels of expression in CF, particularly in the S2 and S3 phases. The genes in Cluster 8 were highly expressed throughout the S2 and S3 phases of CF, and their activities were mostly associated with energy metabolism, such as carbon metabolism, cysteine and methionine metabolism, and the citrate cycle. Clusters 9 and 12 are most active during the S3 and S2 periods, respectively, of the CF. In addition, the genes in this pathway are intimately associated with floral organ abscission, cutin production, and lignin synthesis. Changes in these genes may be among the primary causes of CF early flower abscission. In addition, Cluster 12 genes were mostly involved in amino sugar and nucleotide sugar metabolism, the MAPK signaling pathway, and the metabolism of propanoate. This suggests that CF’s metabolic activity is still active throughout the S2 stage. In conclusion, module clustering analysis revealed the primary functional module of flower abscission difference between CF and CHF: Cluster 9, which regulates CF cell wall and flower abscission through a significant number of genes.

### ABA biosynthesis and expression changes of response genes

**Figure 4:**
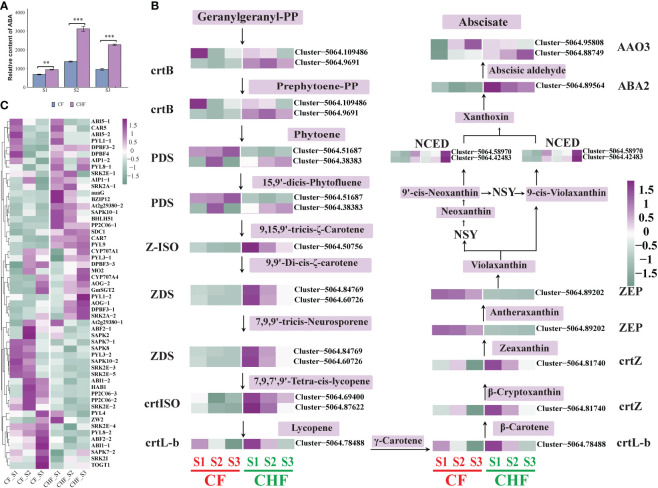
Alteration in gene expression associated with ABA biosynthesis and signal transduction in CF and CHF. **(A)** Variations in the ABA content of CF and CHF receptacles. **(B)** Variations in ABA biosynthesis structural gene expression. **(C)** Changes in gene expression associated with ABA signal transduction and response genes related to ABA. **P < 0.01 and ***P < 0.001.

Analysis of the targeted hormone metabolome revealed substantial variation in ABA content between CF and CHF **(** **Figure 4A** **)**, indicating a significant difference in ABA accumulation between CF and CHF. To further explore the function of ABA accumulation, we compared the principal structural genes in the carotenoid pathway involved in ABA production **(** **Figure 4B** **).** Eleven genes involved in the production of ABA were discovered. In CF, 15-cis-phytoene desaturase (PDS), beta-carotene hydroxylase (crtZ), zeaxanthin epoxidase (ZEP), abscisic-aldehyde oxidase (AAO3), and more structurally important genes were upregulated. PDS was more strongly expressed in the S3 stage, while AAO3 was likewise significantly expressed in the S3 stage. This might suggest that PDS, crtZ, ZEP, and AAO3 are the primary structural genes that regulate ABA production in CF.

In addition to ABA biosynthesis structural genes, we also evaluated the expression of genes involved in ABA signal transmission and other response factors. Among them were 46 genes associated with ABA signal transduction and 20 genes associated with ABA responsiveness and function. The majority of genes involved in ABA signal transduction were upregulated in CF, particularly during the S2 stage **(** **Figure 4C** **)**. The ABA response-related genes of CF were mostly active during the S3 stage. ABF2, SAPK2, SAPK7, SAPK8, SAPK10, ABI1, HAB1, PP2C06, SRK2E, and PYL3 were particularly upregulated in the S2 stage of CF, while PYL4, PYL8, SRK2I, TOGT1, and others were specifically upregulated in the S3 stage. However, PYL1, ABI5, AOG, and others were all downregulated in all phases of CF, but they were upregulated in CHF. This suggests that ABF2, SAPK2, SAPK7, SAPK8, SAPK10, ABI1, HAB1, PP2C06, SRK2E, PYL3, PYL4, PYL8, SRKI, TOGT1, etc., are positive regulators of CF flower abscission, while PYL1, ABI5, AOG, etc., are negative regulators.

### Hormone-related genes that may be involved in abscission

**Figure 5:**
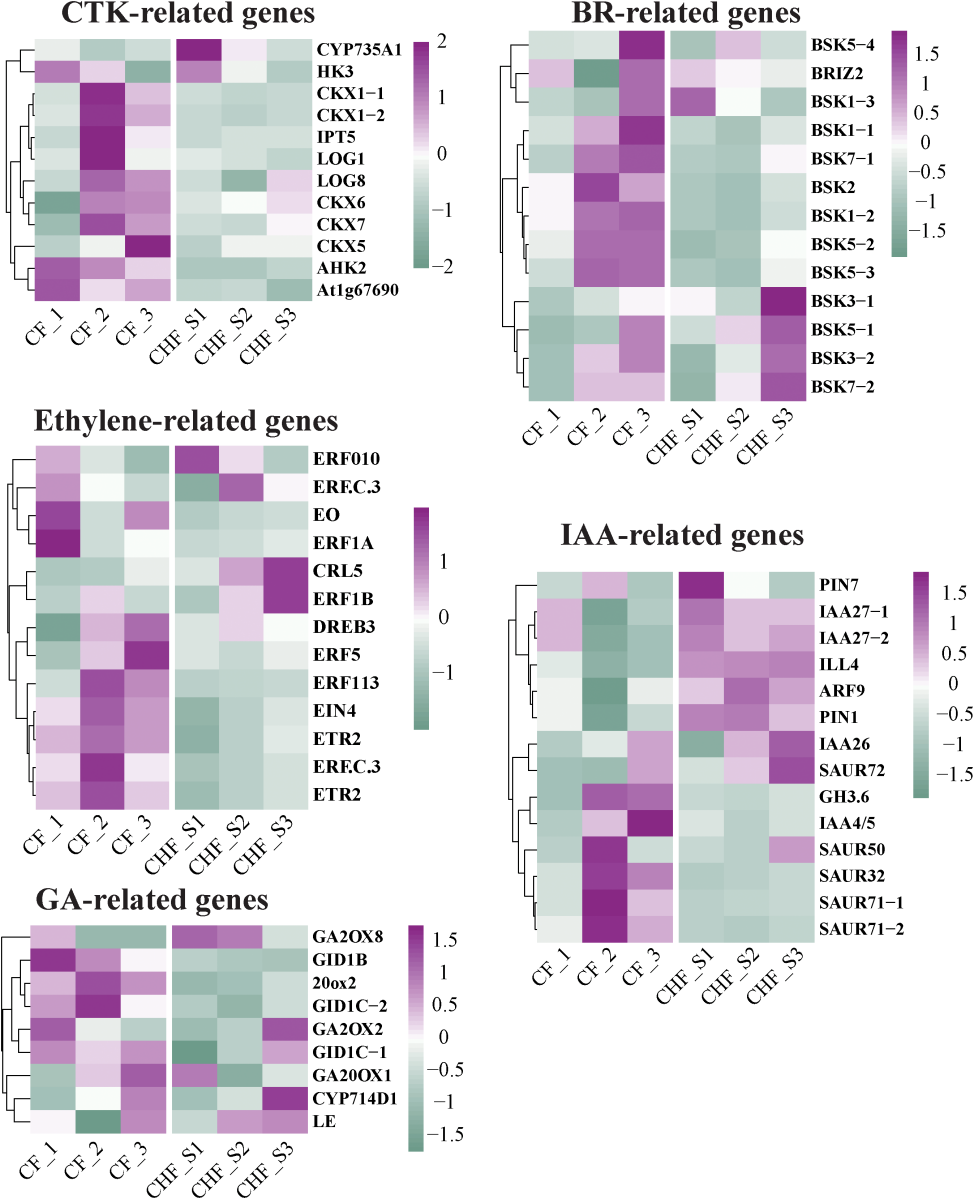
Changes in the differential expression of hormone-related genes in CF and CHF, including CTK, BR, IAA, ethylene, and GA.

Flower abscission in plants is a physiologically complex process regulated by auxins, gibberellins (GAs), cytokinins (CKs), abscisic acid (ABA), ethylene, and brassinolides (BRs). In addition to ABA, the transcriptome analysis found five plant endogenous hormone-related genes that varied during flowering/fruit setting between CF and CHF. These genes included 19 GA-related genes (GRGs), 17 ethylene-responsive genes (ERGs), 14 BR-related genes (BRGs), 20 auxin-responsive genes (AURGs), and 14 CTK-related genes (CRGs) **(** **Figure 5** **).** The majority of CRGs, AURGs, ERGs, GRGs, and BRGs were strongly expressed in CF, with GRGs and CRGs being the most prominent. These genes all seem to be positive regulators of CF flower abscission. This group consists of GID1B, GID1C, GA20OX1, CKX1, CKX5, CKX6, and CKX7. These CRGs and GRGs were characterized as positive regulators of flower abscission in CF because their expression levels increased dramatically throughout the S2 and S3 phases of flower development. Nine ERGs of CF exhibited strong expression in the S2 and S3 phases, whereas the other ERGs exhibited high expression in the S1 stage. The most significant alterations were observed in ERF5, DREB3, ERF113, EIN3, and ETR2, suggesting that these genes are among the most key regulators of flower initiation in CF. Furthermore, we discovered that AURGs such as SAUR32, SAUR50, SAUR71, IAA4, BSK1, BSK2, BSK5, BSK7, and other BRGs displayed distinct high expression in CF, causing us to speculate that they may be possible hormone-related genes controlling CF flower abscission. Furthermore, hormone-related genes were verified by qRT-PCR, and the results showed that the results of qRT-PCR and RNA-seq were consistent (**Figure S2**).

### Transcription factors may be involved in abscission

**Figure 6:**
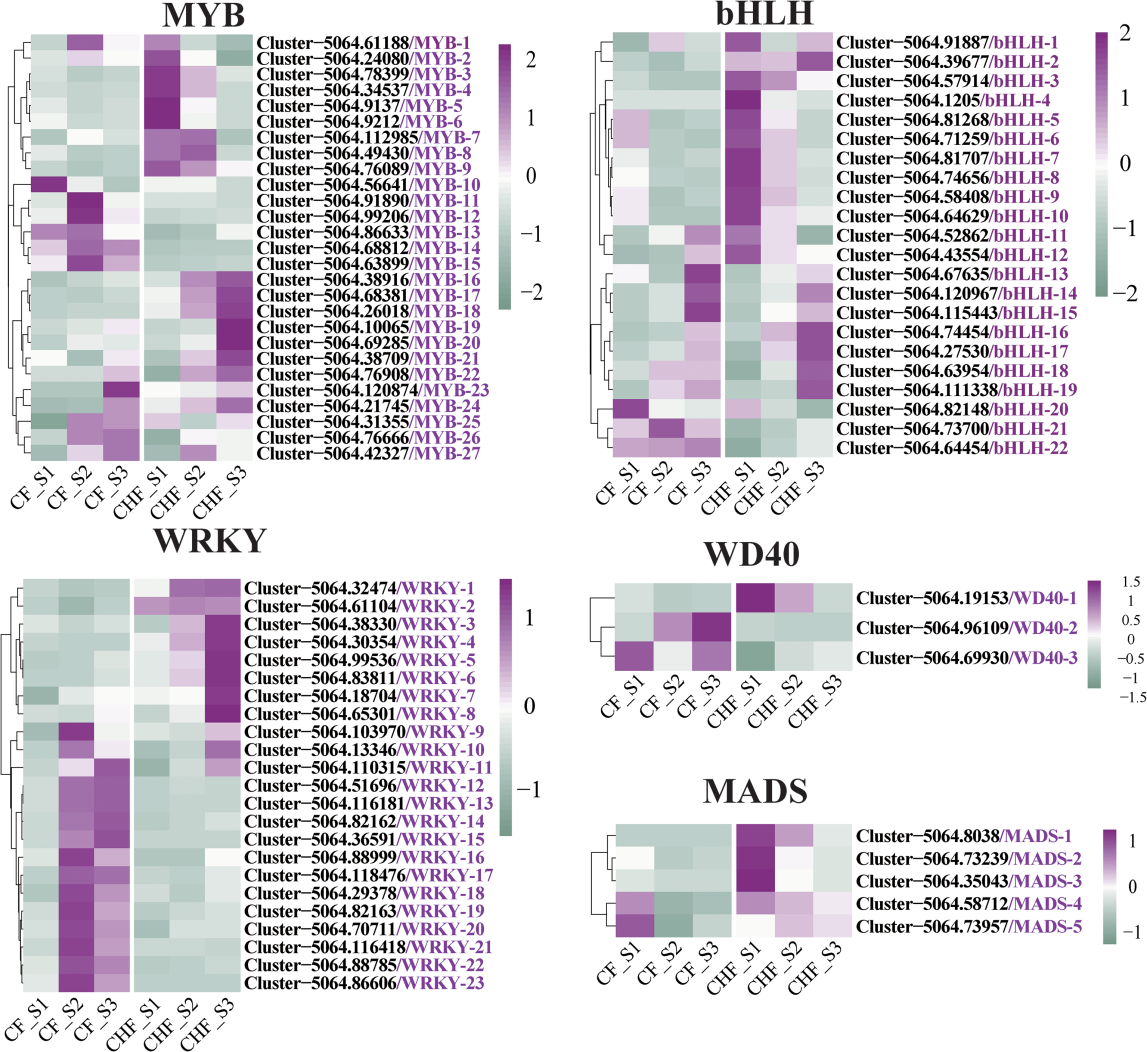
Screening of transcription factor family regulators differentially expressed in CF and CHF. Among them are WRKY, MYB, bHLH, WD40, and MADS.

Several transcription factors may also be connected with the occurrence of plant abscission and bloom development. Identification investigation revealed MYB, WRKY, bHLH, MADS, and WD40 as the primary transcription factors that distinguished CF from CHF. A total of 27 MYB, 21 bHLH, 23 WRKY, 5 MADS, and 3 WD40 members of the transcription factor families were discovered **(** **Figure 6** **)**. The majority of WRKY transcription factor family members, including cjWRKY9-cjWRKY23, were significantly expressed in the S2 and S3 stages of CF, while the other WRKY transcription factors were substantially expressed in the CHF stage. This shows that cjWRKY9-cjWRKY23 may serve as crucial positive regulators of flower abscission in CF, while cjWRKY1-cjWRKY8 may be associated with floral maintenance and may be negative regulators of abscission. In addition, the majority of the bHLH and MYB transcription factor families exhibited upregulated expression in CHF, mostly during the S1 and S3 phases of CHF development. This suggests that the MYB and bHLH transcription factor families may operate primarily as negative regulators of abscission. Five MADS families were highly upregulated throughout the S1 stage of CHF flower development, indicating that they may also be involved in floral formation and maintenance.

## Discussion

This is the first in-depth investigation of the process of abscission in camellias focusing on flower and fruit abscission (Wei et al., 2017; Hu et al., 2021; Zhang et al., 2022). Our approach enables us to investigate not only the genes involved in the molecular processes of abscission (in the receptacle) but also the potential causes of abscission. During flower development, CF and CHF differ significantly from one another. The study revealed that CF and CHF have distinct flower development phases. HCHF exhibited rapid growth and maturity during the S2 stage, while CHF began flower development during the S3 stage after the S2 stage. The quick flower maturity of CF at the S2 stage may result in its simple detachment, while the later flower growth and retention capability of CHF allow for a prolonged blooming time.

In the current work, RNA-seq analysis indicated bidirectional changes in the expression of cell wall-related genes, which may be involved in both the continuing abscission process and the growth of undescended organs (Merelo et al., 2017; Wu et al., 2021). Pathways associated with cell wall formation, including cell wall biogenesis, galactose and mannose metabolism, aminoglycan, pectin synthesis, and other related genes, exhibited divergent patterns at various phases of CHF and CF (Wilmowicz et al., 2021; Gupta et al., 2021). Cell wall production is accompanied by active cell division; several cell wall-related genes may play highly specialized roles in cell wall modification, and some of these gene products may be essential for the optimal function of other genes. Therefore, there is very active transcription of cell wall-related genes in the receptacle, and variations in these cell wall-related genes are predictive of active flower developmental phases. A substantial number of upregulated genes in CF were identified to be associated with floral organ abscission, cutin production, and lignin synthesis (Merelo et al., 2017; Qiu et al., 2020). This indicates that the AZ region of CF may acquire a substantial quantity of lignin. Previous research has shown that lignin accumulation plays a significant role in flower and fruit abscission and that lignin buildup on a huge scale may expedite abscission cell development and keratinization (Su et al., 2019; Feng et al., 2021). Data from RNA-seq reveal that CF has distinct and highly expressed functional modules associated with lignin production and keratinization during flower development, which may be a significant factor in its ease of abscission.

Calcium (Ca^2+^) is necessary for numerous biological activities. In addition to its essential function in maintaining the structural integrity of cell walls and membrane systems, it has been found to operate as an intracellular regulator in several areas of plant growth and development, including cell division and elongation and fruit formation. The Ca^2+^ deficit produced by the downregulation of genes producing Ca^2+^ ion transporters may be an indicator of apple lateral fruit decay, and the Ca^2+^ signaling system is essential for yellow lupin pod abscission, according to previous studies (Glazińska et al., 2019; Mo et al., 2022). In addition, genes involved in the Ca^2+^ signaling pathway were discovered to be downregulated in CF, which may be one of the causes of its loss. In addition, genes involved in pollen identification were dramatically downregulated in HCHF, while they were robustly expressed in the S3 stage of CHF. Pollen recognition is essential to the regular growth of flowers, and unfertilized blooms may be more susceptible to abscission (Ito and Nakano, 2015; He et al., 2020). Low expression of genes involved in pollen recognition may potentially be a risk factor for CF prone to abscission, according to our findings.

Hormones in plants regulate abscission in a very complicated and exact manner. ABA synthesis-related structural genes and ABA signaling-related genes were discovered to be increased in CF, which explains the accumulation of ABA in CF. ABA also influences the production of AZ in the receptacle and facilitates abscission ( Garner and Lovatt, 2016; He et al., 2020). PYL3, PYL4 and PYL8 are all ABA receptors (Santiago et al., 2009). PYL suppresses the activity of group-A protein phosphatases type 2C (PP2Cs) in an ABA-independent manner, but functions more effectively when ABA is active (Zhang et al., 2013). PYL promotes the interaction between ABA and auxin signaling to regulate lateral root development and is essential for ABA-mediated repression of lateral root growth (Parwez et al., 2022). The increased expression of PYL3, PYL4, and PYL8 by CF throughout the S2 and S3 phases of floral development may have a direct relationship with their loss. In addition, plants that overexpressed PP2C06 demonstrated dramatically decreased fertility and severe preharvest germination, which may result in the failure of floral development and the subsequent loss of flowers. Moreover, SRK2E, SAPK8, and SAPK10 are implicated in the ABA-dependent plant stress response (Vahisalu et al., 2010; Li et al., 2015), and the high expression of these genes may make CF more susceptible to environmental influences.

Cytokinins are involved in the control of cell division and growth, while ethylene is hypothesized to mediate the effects of CTK on abscission (Gundesli et al., 2020). Cytokinins influence apical dominance, axillary bud formation, and leaf withering in addition to their primary roles in cell proliferation and differentiation. At the S2 and S3 phases, a large number of CRGs and ERGs were considerably upregulated in CF, and these positive regulators may interact to speed up the division and separation of abscisic cells in the AZ area. The increased expression of CKX1, CKX5, CKX6, and CKX7 in the S2 and S3 phases of CF may reflect the accumulation of CTK in CF. Accumulation of CTK may result in rapid cell division and the development of abscission cells at the receptacle.

Gibberellin is a hormone involved in cell expansion, fruit setting, and growth, and GRG expression was dramatically upregulated during all phases of CF (Zhou et al., 2021). GA20OX1 is a crucial oxidase in gibberellin biosynthesis, and plants that overexpress GA20OX1 have excessive quantities of bioactive gibberellin and an overgrowth phenotype characterized by enhanced internode elongation and leaf sheath length (Oikawa et al., 2004). As soluble gibberellin (GA) receptors, GID1B and GID1C influence root growth, seed germination, and flower development (Ge and Steber, 2018). The increased expression of GA20OX1, GID1B, and GID1C in CF may contribute to the accumulation of GA in CF and enhance the rapid growth of flowers in CF. A flower’s blossoming time may be reduced if its growth is accelerated, resulting in early flower fall. Studies indicate that auxin may function as a signal from a fertilized ovule, stimulating GA production and initiating fruit development. We hypothesize that a similar circumstance exists in CF and that the synergy between GA and IAA is likewise a risk factor for CF abscission.

ERF5, ERF113, EIN3, and ETR2 are also essential genes in the ethylene response pathway. They may directly regulate gene transcription or serve as ethylene receptors to govern the environmental sensitivity of plants (Dutta et al., 2022; Zhu et al., 2022). As a vital plant hormone, ethylene has the ability to accelerate fruit ripening. We propose that ethylene affects floral organ senescence and separation through ERF5, ERF113, EIN3, and ETR2 in CF. We discovered that SAUR50 was likewise uniquely elevated in CF. SAUR50 provides a mechanistic link between auxin and plasma membrane H+-ATPases (PM H+-ATPases, e.g., AHA1 and AHA2) and triggers PM H+-ATPase activity by promoting C-terminal autoinhibitory domain phosphorylation as a result of PP2C-D subfamily of type 2C phosphatase inhibition, resulting in acidification of the apoplast, and the facilitation of solute imbalance between cell division and development in the torus area may be one of the primary causes of abscission region creation (Ren and Gray, 2015). SAUR50 might influence AZ formation in CF by controlling local cell proliferation, hence influencing flower abscission.

## Data availability statement

The datasets presented in this study can be found in online repositories. The names of the repository/repositories and accession number(s) can be found below: https://www.ncbi.nlm.nih.gov/, PRJNA889986.

## Author contributions

YFC: Conceptualization. YSC: Formal analysis. YFC and JW: Funding acquisition. YFC and JM: Investigation. YFC, JM, YSC, MT, and ZS: Methodology. JW: Supervision. YFC and JW: Writing – review & editing. All authors contributed to the article and approved the submitted version.
