# Supplementary material for: Transcriptome and targeted hormone metabolome reveal the molecular mechanisms of flower abscission in camellia
Source: Front Plant Sci. 2022 Dec 22;13:1076037. doi: 10.3389/fpls.2022.1076037 (PMC9813748; doi:10.3389/fpls.2022.1076037)
Supplement: Supplementary file 1 [file DataSheet_1.docx]

**Transcriptome and Targeted Hormone Metabolome Reveal the Molecular Mechanisms of Flower Abscission in Camellia**

Yanfei Cai^1, 2, †^, Jing Meng^3, †^, Yinshan Cui ^4^, Min Tian^1, 2^, Ziming Shi^1, 2^ and Jihua Wang^1, 2, *^

1 Flower Research Institute of Yunnan Academy of Agricultural Sciences, Kunming, Yunnan, China;

2 National Engineering Research Center for Ornamental Horticulture, Kunming, Yunnan, China

3 College of Horticulture and Landscape, Yunnan Agricultural University, Kunming, Yunnan, China

4 Yunnan Pulis Biotechnology Co. Ltd., Kunming, Yunnan, China

† These authors contributed equally to this work and share first authorship.

Jihua Wang *

wjh0505@gmail.com; Tel.: +86-138-8882-2307

Supplementary Table 1 The primer sequences for qRT-PCR

| Gene ID | Gene name | Forward | Reverse |
| --- | --- | --- | --- |
| Cluster-5064.72431 | actin | GCAGAAGCAAGTTATTGTGGAA | CATTCGTCAGCAGGAAGACTA |
| Cluster-5064.69166 | PIN | AAGTGAGAGTTGCGGTATCT | GCTTTGCCTCTGTTCTTGTT |
| Cluster-5064.67852 | PP2C | GGAGAAGATGGCGACAAG | TTACATTGTCAGCACTCCG |
| Cluster-5064.83744 | ETR | ATGAATGCCTTAGTGCTCTTG | GCAAACATCTATCCCACACAT |
| Cluster-5064.70628 | SAUR | AAGCCGAGGAAGAGTACG | GAGCCGAGTTACAGGACT |
| Cluster-5064.81781 | PP2C | TTGAGAGGGCTGACAATGAG | GGCAACCTGATAGAACCACA |
| Cluster-5064.65114 | EO | AAGAGGGACAGACGGTTT | TTCGGTGTGCTAGTTGTTG |

Supplementary Table 2 The assembly and annotation statistics

| Unigenes | Number (Percentage) |
| --- | --- |
| total number of unigenes | 138,557 |
| min length | 301 |
| mean length | 1033 |
| median length | 687 |
| max length | 14,764 |
| N50 | 1,463 |
| N90 | 455 |
| **BUSCO assessment results** | |
| Complete(single) | 756(52.5%) |
| Complete(duplicated) | 48(3.3%) |
| Fragmented | 315(21.9%) |
| Missing | 321(22.3%) |
| **Annotation statistics** | |
| NR | 67555 (48.75%) |
| NT | 98112 (70.8%) |
| KO | 19607 (14.15%) |
| SwissProt | 39642 (28.61%) |
| PFAM | 40686 (29.36%) |
| GO | 40676 (29.35%) |
| KOG | 9628 (6.94%) |
| all databases | 5379 (3.88%) |

Supplementary figure


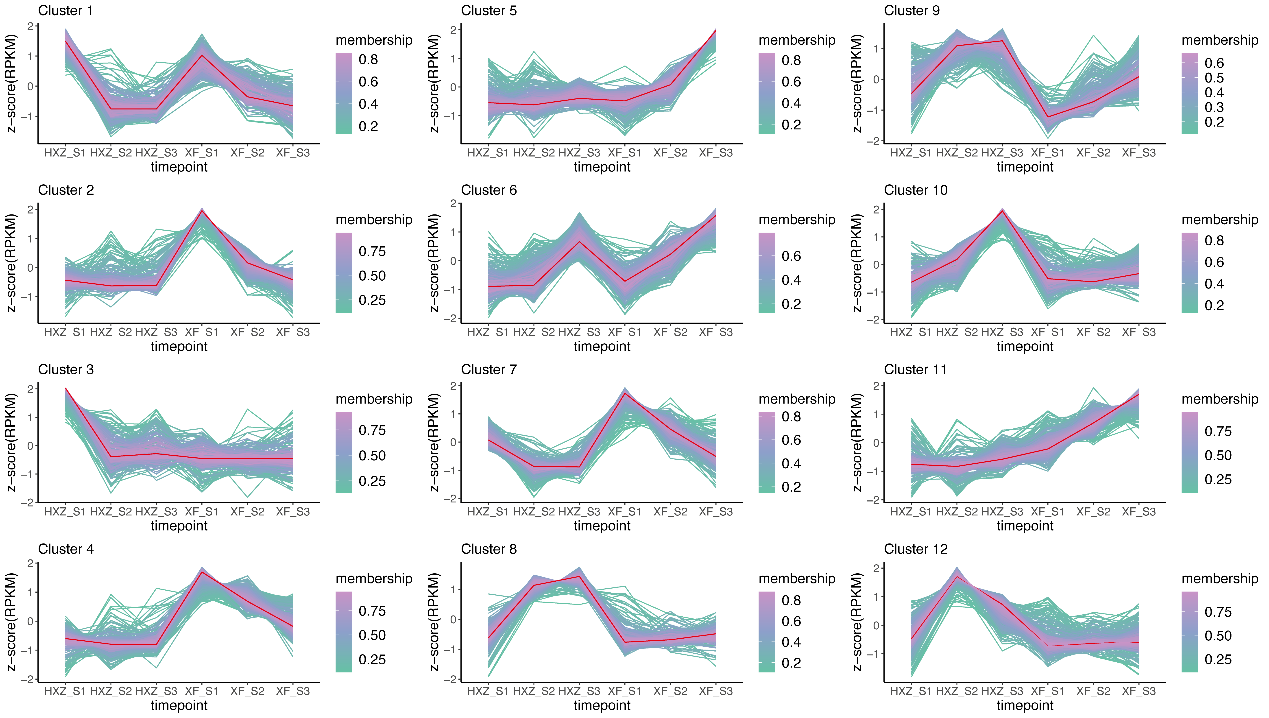


Figure S1 All DEG were clustered into 12 subgroups by K-means cluster analysis.


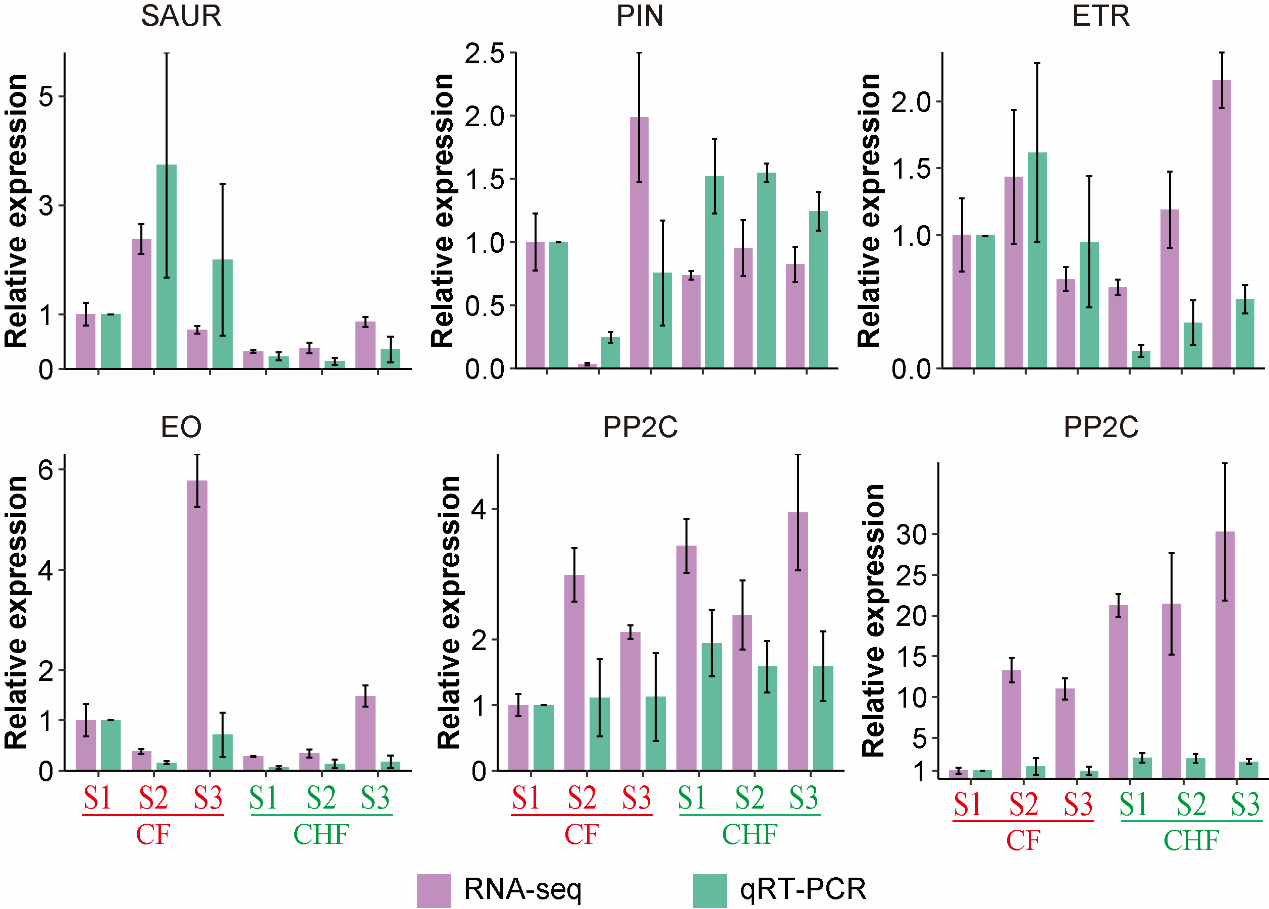


Figure S2 The qRT-PCR results confirm the transcriptome expression of the RNA sequencing.
